# Acetylcholineestarase-Inhibiting Alkaloids from *Lycoris radiata* Delay Paralysis of Amyloid Beta-Expressing Transgenic *C. elegans* CL4176

**DOI:** 10.1371/journal.pone.0063874

**Published:** 2013-05-13

**Authors:** Lijuan Xin, Ritupriya Yamujala, Yuehu Wang, Huan Wang, Wen-Hsuan Wu, Michael A. Lawton, Chunlin Long, Rong Di

**Affiliations:** 1 College of Life and Environmental Sciences, Minzu University of China, Beijing, China; 2 New Brunswick Graduate School, Rutgers University, New Brunswick, New Jersey, United States of America; 3 Key Laboratory of Economic Plants and Biotechnology, Kunming Institute of Botany, Chinese Academy of Sciences, China; 4 Department of Plant Biology and Pathology, School of Environmental and Biological Sciences, Rutgers University, New Brunswick, New Jersey, United States of America; Oregon Health & Science University, United States of America

## Abstract

The limited symptom relief and side effects of current Alzheimer’s disease (AD) medications warrant urgent discovery and study of new anti-AD agents. The “cholinergic hypothesis” of AD prompts us to search for plant-derived acetylcholineesterase (AChE) inhibitors such as galanthamine that has been licensed in Europe for AD treatment. We used the unique amyloid β-expressing transgenic *C. elegans* CL4176, which exhibits paralysis when human Aβ_1–42_ is induced, to study two natural benzylphenethylamine alkaloids isolated from *Lycoris radiata* (L’ Her.) Herb, galanthamine and haemanthidine, and their synthetic derivatives 1,2-Di-*O*-acetyllycorine and 1-*O*-acetyllycorine for their anti-paralysis effects. Our data indicate that these *Lycoris* compounds effectively delay the paralysis of CL4176 worms upon temperature up-shift, and prolong the lives of these transgenic worms. *Lycoris* compounds were shown to significantly inhibit the gene expression of *ace-1* and *ace-2.* Additionally, the *Lycoris* compounds may modulate inflammatory and stress-related gene expressions to combat the Aβ-toxicity in *C. elegans*.

## Introduction

With more than 20 million cases worldwide, Alzheimer’s disease (AD) has been named the most common progressive neurodegenerative disease [1]. AD is characterized by cerebral degeneration, neuronal cell death, and the hallmark accumulation of 40–42 amino acid amyloid-β (Aβ) in plaques [2] and tau tangles in the affected brain nerve cells [1]. According to the “amyloid hypothesis” [3], [4], Aβ is produced by the cleavage of the ubiquitous amyloid precursor protein (APP) and the imbalance between the production and the clearance of Aβ in central nervous system, leads to neuronal degeneration. Up to now, there is no cure for this debilitating disease. The widely accepted “cholinergic hypothesis” of AD proposes that a serious loss of cholinergic function in the central nervous system is associated with the development of cognitive symptoms [5]. A number of acetylcholinesterase (AChE) inhibitor drugs, including tacrine, donepezil, and galantamine, have been developed and are used to restore the normal cholinergic function for synaptic transmission in the central nervous system of AD patients [6]. These drugs, which are moderately effective in alleviating the symptoms of AD, have a number of side effects, including gastrointestinal upsets [6], [7]. Other drugs used to treat AD patients, such as memantine, attempt to balance the cholinergic pathway by attenuating the function of N-methyl-D-aspartate (NMDA) receptors. Like AChE inhibitors, memantine offers only modest AD symptom relief along with gastrointestinal side effects [8]. As the world population continues to live longer and AD patient numbers steadily rise, the need for new, effective and safer pharmacological agents for AD therapy becomes increasingly pressing.

In recent years, we have been working on the isolation of active anti-AD compounds from traditional Chinese herbs. *Lycoris* Herb. is a member of Amaryllidaceae and a genus endemic to East Asia. *Lycoris radiata* (L’ Her.) Herb has been used in traditional Chinese medicine to treat sore throat, rheumatoid arthritis and snake bites. [9] *Lycoris* plants are rich in benzylphenethylamine alkaloids such as galanthamine, lycorine, lycoramine and lycorenine [10], [11]. We have previously reported the isolation of fifteen known benzylphenethylamine alkaloids in 2011 from the bulbs and flowers of *Lycoris radiata* [9]. Some alkaloids were first reported from *Lycoris* Herb. and some were first isolated from *L. radiata*, including haemanthidine. Both galantamine and lycoramine from *L. radiata* have been reported to have anti-AChE activities [12]. Being orally bio-available and stimulative to nicotinic acetylcholine receptors (nAchR) [13], galanthamine is licensed in Europe for AD treatment and is well tolerated by AD patients [12].

Besides the AChE inhibitory effects, galanthamine has been found to modulate inflammation by attenuating TNF-α (tumor necrosis factor-alpha) and NO (nitric oxide) release through the α7 nAChR [14] and p44/42 MAPK (mitogen-activated protein kinase) pathway in murine microglia [13]. Another AChE inhibitor, donepezil, has also been shown to decrease cytokine (oncostatin M, interleukin-1β and interleukin-6) levels in AD patient lymphocytes [15]. Tacrine, donepezil and huperzine, all AChE inhibitors, have been demonstrated to prevent hydrogen peroxide-induced cell death and Aβ peptide-induced oxidative cell death [16]–[18]. Since inflammatory process and oxidative damage have been implicated in neurodegenerative diseases, any AChE inhibitory agent with the additive anti-inflammatory and/or anti-oxidative effects would be expected to be superior for AD treatment [6], [7], [15].

Transgenic *Caenorhabditis elegans* (*C. elegans*) models have been established for Alzheimer’s disease since 1995 [19]. Nematode disease models have been used to study the mechanisms of AD toxicity [20] and to test the efficacies of drugs and nutri-supplements. By using transgenic CL4176 worms, which express the human Aβ_1–42_ in muscle tissues under a temperature-inducible system [20], it was reported that soy isoflavone glycitein could protect worms from Aβ-induced toxicity and this protection was credited to the anti-oxidative activity of glycitein [21]. *Ginkgo biloba* extract EGb761 and ginkgolides were shown to suppress the Aβ-induced pathological behaviors of several different Aβ-transgenic *C. elegans*, not by reducing oxidative stress but rather by modulating Aβ oligomeric species [22]. Arya *et al*. used transgenic *C. elegans* strains CL2006, which constitutively expresses Aβ in the body wall muscles, and CL2355, which has inducible neuronal Aβ expression, to show that reserpine (an FDA approved antihypertensive drug) could ameliorate Aβ toxicity [23].

In this study, we used transgenic *C. elegans* CL4176 to evaluate the Aβ toxicity- inhibitory effect of galanthamine and haemanthidine purified from *Lycoris radiata* (L’ Her.) Herb. and their derivatives 1,2-Di-*O*-acetyllycorine and 1-*O*-acetyllycorine. We demonstrate that these *L. radiata* compounds strongly inhibit Aβ toxicity and prolong the lifespan of CL4176 worms. Attenuation of Aβ toxicity in this model system mostly results from the inhibition of acetylcholineesterase gene expression. Modulation of inflammation and stress-related genes may also contribute to the anti-Aβ toxicity of *Lycoris* compounds. Our study indicates that Aβ transgenic CL4176 nematodes can be efficiently used to screen for AChE inhibitors.

## Results

### Isolation and Synthesis of *L. radiata* Compounds

From the bulbs and flowers of *L. radiata*, we obtained fifteen known alkaloids [9]. Galanthamine and haemanthidine were selected for further study. According to a previous report, 1-*O*-acetyllycorine possesses potent activity against electric eel acetylcholinesterase (eeAChE, IC_50_ = 0.96 µM) [24]. Therefore, we also synthesized this compound and its analogue 1,2-di-*O*-acetyllycorine and used both in the following studies.

### Natural and Synthetic *L. radiata* Compounds Delayed Paralysis of Aβ-Transgenic *C. elegans* CL4176 Nematodes

**Figure 1:**
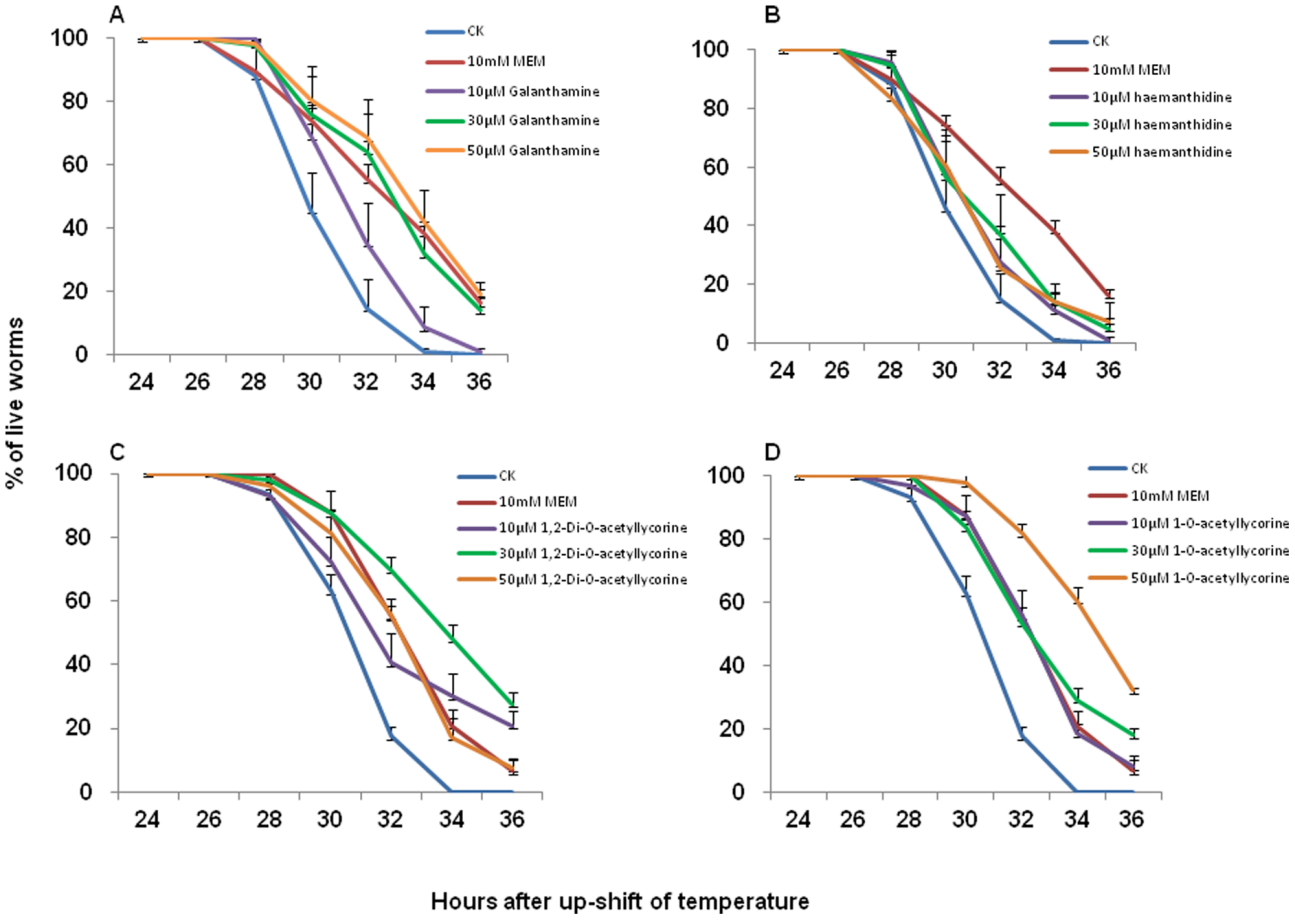
Time course of the paralysis assay of CL4176 *C. elegans*. Synchronized worms were hatched and fed on compound-containing NGM medium for two days at 16°C. The temperature was then shifted to 23°C. The survival rate (% of live worms) was recorded and plotted against the hours post temperature shift. The worms were treated with (**A**) galanthamine, (**B**) haemanthidine, (**C**) 1,2-Di-*O*-acetyllycorine and (**D**) 1-*O*-acetyllycorine at 10, 30 and 50 µM concentrations. In comparison, one group of worms were not treated (the non-treated control sample, CK) and the other group ware treated with 10 mM memantine. Three independent experiments were carried out to give the average rates for worm survival and to calculate standard deviations.

CL4176 worms were synchronously hatched and raised on NGM plates containing OP50 bacteria and compounds at 16°C. After two days, the temperature was shifted to the permissive 23°C to induce expression of the Aβ_1–42_ peptide. We consistently observed that 26 hours after the temperature up-shift, non-treated worms started to become paralyzed and die due to the expression of human Aβ_1–42_. In order to compare the efficacy of anti-Aβ activity of *L. radiata* compounds with memantine, the current medication used to lessen symptoms in AD patients, we included memantine as one of the experimental treatments in these studies. While memantine has been shown to be effective in delaying the paralysis of CL4176 upon temperature shift to 23°C, the concentration used in that study was not reported [25]. We tested different concentrations of memantine in the paralysis assay with CL4176 and found that it was effective only in the millimolar range (data not shown). Consequently, we used 10 mM of memantine as a reference throughout these studies. We showed that galanthamine could reduce the mortality of CL4176 compared to the control (CK) worms which were not treated with any compound (Fig. 1A). The control worms reached 100% mortality 34 hr post temperature up-shift. In contrast, 8% of worms exposed to 10 µM galanthamine were still alive at 36 hr. The worm survival rates were further increased to 32% and 42% by 30 µM and 50 µM of galanthamine 34 hr post temperature up-shift. Our data showed that the anti-paralysis effect of 50 µM galanthamine was greater than that of 10 mM memantine. We further demonstrated that 50 µM galanthamine could prolong the lifespan of CL4176 up to 40 hr.

Our data also showed that haemanthidine, which we isolated for the first time from *L. radiata*, could reduce the mortality of CL4176, with 11%, 14% and 14% worms still alive 34 hr after the temperature up-shift at concentrations of 10 µM, 30 µM and 50 µM (Fig. 1B). At these same concentrations, haemanthidine appeared to be less effective at delaying the paralysis of CL4176 nematodes as compared to galanthamine.

The first synthetic alkaloid 1,2-Di-*O*-acetyllycorine at 50 µM was shown to be comparable to10 mM memantine in preventing paralysis of CL4176 worms expressing the Aβ_1–42_ peptide (Fig. 1C). At 30 µM, however, 1,2-Di-*O*-acetyllycorine increased the worm survival rate to 48% 34 hr post temperature up-shift, which was similar to the effect of 50 µM galanthamine (Fig. 1A).

The second synthetic alkaloid 1-*O*-acetyllycorine exhibited similar anti-paralysis effects in CL4176 worms as 10 mM memantine, and was comparable to 30 µM galanthamine at the same concentration (Fig. 1D). However, 50 µM 1-*O*-acetyllycorine increased the worm survival rate of CL4176 to the greatest level (61% alive), compared to all the compounds tested.

Our data indicate that the new alkaloid haemanthidine from *L.* *radiata* and our synthetic alkaloids 1,2-Di-*O*-acetyllycorine and 1-*O*-acetyllycorine could reduce Aβ-toxicity with similar potency to the known AChE inhibitor galanthamine. We have shown our *Lycoris* compounds were effective at the micromolar concentration range versus the millimolar concentration range required for the current AD medication memantine. Therefore, 50 µM for all *Lycoris* compounds and 10 mM for memantine were subsequently used in the following experiments.

### *L. radiata* Compounds Slightly Reduced the Levels of Aβ in Transgenic *C. elegans* CL4176

Since the paralysis and the subsequent death of CL4176 are caused by the Aβ toxicity, naturally we wanted to examine if our *Lycoris* compounds would have any direct effect on the expression of Aβ ˜Transgenic *C. elegans* CL4176 expresses Aβ in the worm muscle cells [19]. It has been shown that CL4176 worms actually do not form Aβ plaques in the worm bodies. Rather, Aβ is expressed mostly in a soluble form [25], [26]. We used the fluorescent thioflavin T stain, which detects non-aggregated proteins more specifically than thioflavin S [27], to quantify the levels of Aβ in CL4176 worms treated with *L. radiata* compounds. Pure synthetic Aβ obtained from Sigma (cat. # A9810) was initially used to optimize the assay condition by preparing 0, 1, 5, 10 to 100 µg Aβin 100 µl volume each with 20 µM thioflavin T. It was determined that the Aβ concentration effect could be assessed by our Synergy HT Plate Reader using 440 nm excitation and 482 nm emission. At least 50 worms were collected from each treatment and sonicated. Equal amount of total soluble protein was used for each sample and assayed in triplicates and the experiment was repeated three times. Our results demonstrated that there was no reduction in the level of Aβ by any of the *Lycoris* compounds or by memantine 26 hr after the temperature shift to 23°C. As mentioned above, at 26 hr post temperature up-shift, the worms became paralyzed and started to die. We then repeated the treatments and collected worms 32 hr post temperature up-shift. The thioflavin T assay results showed that the Aβ levels in the galanthamine, haemanthidine, 1,2-Di-*O*-acetyllycorine, 1-*O*-acetyllycorine and memantine treated worms were 91.5% ±4.86 (*p = *0.019), 91.37% ±7.72 (*p = *0.062), 91.23% ±9.26 (*p = *0.088), 93.61% ±1.41 (*p = *0.001) and 98.27% ±1.83 (*p = *0.085), compared to the non-treated worms. These data indicate that galanthamine significantly reduced the Aβ level by approximately 8.5% and the synthetic 1-*O*-acetyllycorine significantly reduced the Aβ level in worms by approximately 6.4% compared to the non-treated worms. In comparison, the data also indicate that memantine did not seem to reduce the Aβ level much.

Quantitative real-time RT-PCR (qRT-PCR) was carried out to assess the capability of *Lycoris* compounds to inhibit the Aβ gene at the transcript level. By using the primers designed for the Aβ transgene and the *F23B2.13* gene encoding an RNA polymerase small subunit as a non-variable endogenous control [28] (Table 1), the relative gene expression of Aβ was assessed by the 2^−ΔΔCt^ method. Our qRT-PCR results from three independent experiments showed that galanthamine significantly reduced the Aβ transgene expression by an average of 2.72-fold (*p*<0.01) compared to the non-treated sample. The results also showed that 1,2-Di-*O*-acetyllycorine reduced the Aβ gene expression by 1.32-fold (*p*<0.05) and 1-*O*-acetyllycorine by 1.3-fold (*p*<0.01). Since it is generally accepted that a 2-fold change in gene expression is considered significant, we can assume the effect of 1,2-Di-*O*-acetyllycorine and 1-*O*-acetyllycorine on Aβ gene expression was negligible. Haemanthidine and memantine, however, did not seem to have any inhibitory effect on the Aβ transgene expression at the transcript level.

**Table 1:** Oligonucleotide primers used in qRT-PCR studies.

Gene	Forward primer	Reverse primer
A**β**	CCGACATGACTCAGGATATGAAGT	CACCATGAGTCCAATGATTGCA
*ace-1*	AGTGGGCTCCTGTTCGAGAA	CCAATAGAAAATCACCATCGACAA
*ace-2*	CAATAATCAACTCATGGGCATCA	TTTTCGCGAGACGAAACGA
*F22E5.6*	TCCCCATACGAAACAACACA	CTCCTCCCAGCTTTTCCACAA
*ZC239.12*	CCAGAAGAATCCCCATACGA	TCCTCCTCCAACTTTTCCAAA
*Y46H3A.D*	GGTGCAGTTGCTTCGAATCTT	TCTTCCTTGAACCGCTTCTTTC
*Y46H3A.E*	AAACAAAATCGGAACATGGATACTT	TGGAGCCTCAATTTGGAGTTTTC
*F23B2.13*	CGCCGAAAATGAAATCAAAC	GGGCGTCGTACACCATCA

### *L. radiata* Compounds Inhibited Acetylcholine Esterase Gene Expression in Aβ-Transgenic *C. elegans* CL4176 Nematodes

To delineate the anti-paralysis mechanisms of natural and synthetic *L. radiata* compounds in Aβ-transgenic *C. elegans* CL4176 nematodes, we investigated the ability of the natural and synthetic *L. radiata* compounds to inhibit the AChE gene expression in Aβ-transgenic *C. elegans* CL4176 nematodes. Total worm RNA was isolated from CL4176 nematodes 26 hr after the temperature up-shift. At this point, the worms had fed for a total of 48 hr on medium containing both the compounds and OP50 bacteria and just started to become paralyzed. Unlike vertebrates with only one AChE gene [29], *C. elegans* and other nematodes have multiple *ace* genes. *C. elegans ace-1* gene is expressed in all body-wall and vulval muscle cells [30] while *ace-2* is expressed almost exclusively in neurons [31]. *ace-3* is expressed in pharyngeal muscle cells and neurons [32]. ACE-1 and ACE-2 account for 95% of the total enzymatic activity, ACE-3 for the remainder 5% and ACE-4′s activity is normally undetectable. Consequently, we focused on the effects of *L. radiata* compounds on gene expression of *ace-1* and *ace-2*.

**Figure 2:**
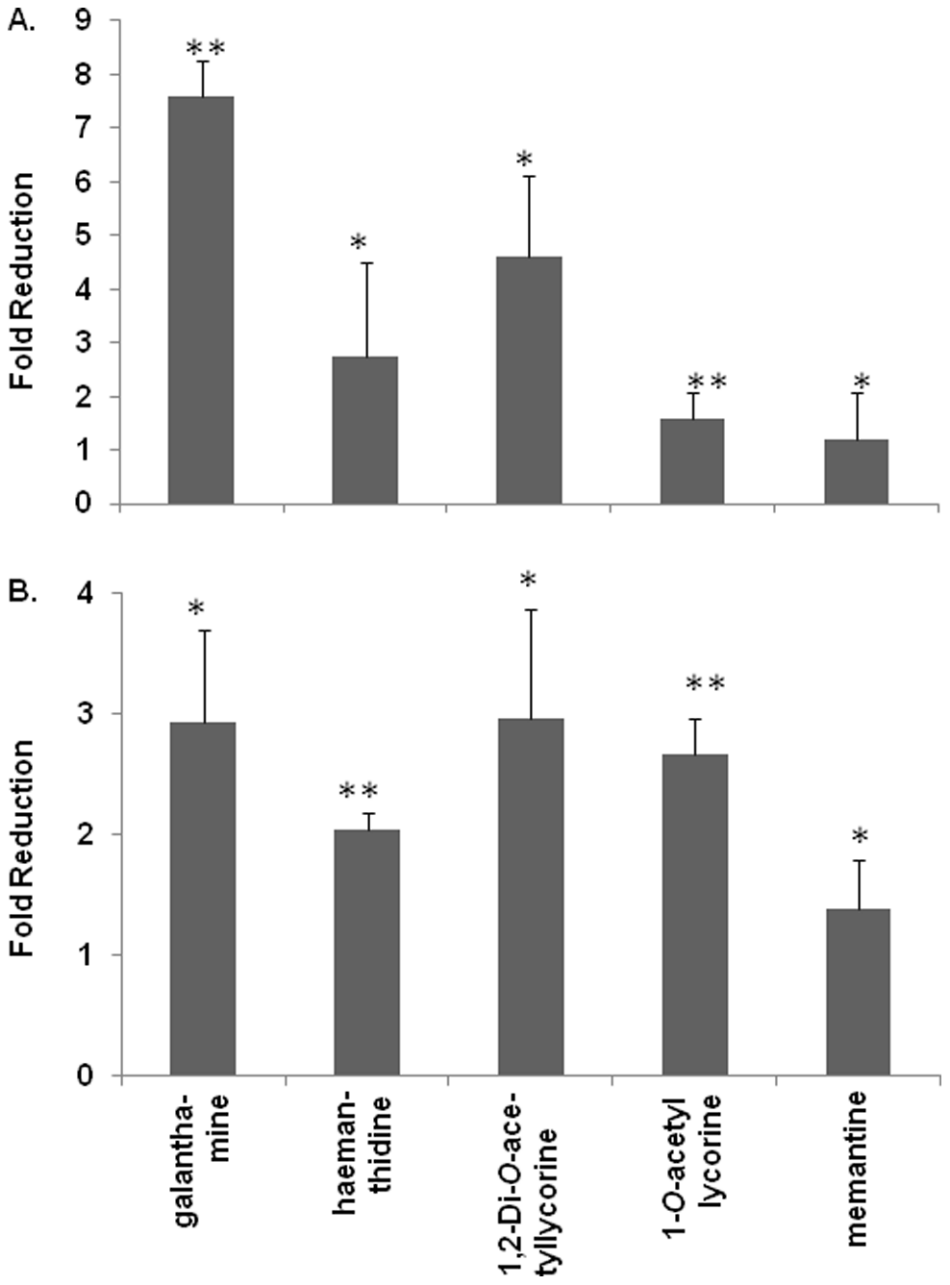
Real-time qRT-PCR analysis of the steady state acetylcholine esterase gene expression in transgenic *C.* elegans. Total RNA was isolated from worms 26 hr post temperature up-shift and subjected to DNase digestion. cDNA was produced using the High Capacity cDNA Synthesis Kit (Life Technologies) with random primers. qPCR was performed with Applied Biosystem SDS7300 instruments and primers specific for *ace-1* (**A**) and *ace-2* (**B**) genes. The gene expression levels were assessed by the 2^−ΔΔCt^ method using *F23B2.13* gene as a non-variable endogenous control. The fold-reductions and standard deviations for gene expression were calculated from three independent experiments by comparison with non-treated samples. The student *t*-test was conducted to assess the statistical significance (*p<0.05; **p<0.01).

To study the steady state expression levels of the *ace-1* and *ace-2* genes, we performed qRT-PCR assays using specific forward and reverse primers for these genes (Table 1) with total nematode RNA isolated from Aβ-transgenic *C. elegans* CL4176. The relative levels of *ace-1* and *ace-2* gene expression were assessed by the 2^−ΔΔCt^ method using the *F23B2.13* gene as a non-variable endogenous control, averaged from three independent experiments. Galanthamine reduced the gene expression of *ace-1* by 7.61-fold, the synthetic 1,2-Di-*O*-acetyllycorine decreased *ace-1* expression by 4.62-fold and haemanthidine decreased *ace-1* expression by 2.73-fold (Fig. 2A). Our results also demonstrated that the synthetic 1-*O*-acetyllycorine reduced the expression of *ace-1* in CL4176 worms by only 1.6-fold and that memantine had no inhibitory effect on *ace-1*. Additionally, we demonstrated that the synthetic 1,2-Di-*O*-acetyllycorine reduced *ace-2* expression by 2.97-fold (*p*<0.05), galanthamine reduced *ace-2* expression by 2.94-fold (*p*<0.05), synthetic 1-*O*-acetyllycorine decreased *ace-2* expression by 2.67-fold (*p*<0.01) and haemanthidine decreased *ace-2* expression by 2.04-fold (*p*<0.01). Memantine had a slightly inhibitory effect, reducing *ace-2* levels by 1.39-fold which was negligible.

### *L. radiata* Compounds Inhibited Inflammation and Stress-Associated Gene Expression Which May Contribute to the Anti-Aβ Paralysis Effect in *C. elegans* CL4176

As inflammation is implicated in Aβ-toxicity and AChE inhibitors have been shown to modulate inflammation to improve the cholinergic health [7], [15], [28], we decided to evaluate the ability of *L. radiata* compounds to reduce the expression of inflammation and stress-associated genes in CL4176 *C. elegans*. Pro-inflammatory genes and cytokines such as TNFα, IL-1 and IL-6 are associated with human neurodegenerative diseases. While *C. elegans* contains homologues of TNFA1P1 (TNFα-induced protein 1), this gene has not been associated with Aβ toxicity in human [28]. However, it has been reported that the treatment of TNFα in human cell cultures exposed to Aβ can protect hippocampal neurons from Aβ toxicity [33]. Link *et al*. have shown that the gene expression levels of two *C. elegans* homologs of human TNFA1P1, *F22E5.6* and *ZC239.12*, were up-regulated in transgenic CL4176 upon temperature up-shift [28]. They also showed that TNFA1P1 expression was increased in AD patient brain tissues [28]. Additionally, Link *et al*. reported that the expression of human αB-crystallin (CRYAB), a well-studied stress-inducible chaperone protein, was enhanced in AD patient brain tissues. Equivalently, the *C. elegans* CRYAB-homologous HSP16 (heat shock protein 16)-2 (*Y46H3A.D*) and HSP16-4 (*Y46H3A.E*) gene expressions were up-regulated in transgenic CL4176 worms following the temperature up-shift [28].

We used the same primers in Link *et al*. [28] (Table 1) to perform qRT-PCR on total RNA isolated from CL4176 26 hr after the temperature shift. We found that galanthamine, haemanthidine and 1,2-Di-*O*-acetyllycorine at 50 µM significantly reduced the gene expression of TNFA1P-homolog *F22E5.6* (*p*<0.01∼0.05), with haemanthidine being the most effective compound with an average of 4.8-fold reduction compared to the non-treated sample (Table 2). 1,2-Di-*O*-acetyllycorine at 50 µM was also shown to affect another TNFA1P1-homolog, *ZC239.12*, whose gene expression was reduced by 5.5-fold (a significance level of *p = *0.006). Additionally, our data indicate that 50 µM haemanthidine reduced the gene expression of two stress-related HSP-16 genes by 1.99- and 2.07-fold, corresponding to a statistical significance values of *p = *0.007, and 0.025, respectively. These results show us that galanthamine, haemanthidine and 1,2-Di-*O*-acetyllycorine may be involved in limiting inflammation and stress-related cellular damage caused by Aβ toxicity in transgenic CL4176 worms. Interestingly, we found that 10 mM memantine could reduce the expression of these four genes related to inflammation and stress in CL4176 *C. elegans* (Table 2).

**Table 2:** Inhibition of inflammation and stress-associated gene expression in CL4176 nematodes.

Gene	Description	Compounds	Average Fold reduction	*P* value	
*F22E5.6*	TNFA1P1 homolog	galanthamine	2.17		0.039*
		haemanthidine	4.8		0.033*
		1,2-Di-*O*-acetyllycorine	1.39		0.012*
		1-*O*-acetyllycorine	1.49		0.152
		memantine	2.78		0.011*
*ZC239.12*	TNFA1P1 homolog	galanthamine	2.11		0.054
		haemanthidine	0.98		0.079
		1,2-Di-*O*-acetyllycorine	5.55		0.0066**
		1-*O*-acetyllycorine	1.41		0.15
		memantine	7.3		0.002**
*Y46H3A.D*	HSP16-2	galanthamine	1.17		0.0048*
		haemanthidine	1.99		0.007**
		1,2-Di-*O*-acetyllycorine	0.99		0.002**
		1-*O*-acetyllycorine	2.06		0.098
		memantine	2.29		0.007**
*Y46H3A*	HSP16-41	galanthamine	0.94		0.009**
		haemanthidine	2.07		0.025*
		1,2-Di-*O*-acetyllycorine	0.77		0.008**
		1-*O*-acetyllycorine	1.81		0.071
		memantine	3.12		0.019*

Average fold-reduction of gene expression was calculated from three independent qRT-PCR analyses by comparing samples from treated and non-treated CL4176 worms collected 26 hr after temperature shift. Student *t*-test was used to calculate *p* values for statistical significance,

* *p*<0.05,

** *p*<0.01.

### Antioxidant Activity did not Contribute to the Anti-Aβ Paralysis of CL4176 Nematodes by *L. radiata* Compounds

Besides Aβ toxicity, oxidative stress has been widely postulated to contribute to the etiology of Alzheimer’s disease [7], [26]. Aβ-transgenic *C. elegans* CL4176 have been shown to be under increased oxidative stress upon the temperature shift to 23°C [21]. We used the dye DCF-DA to measure intracellular levels of H_2_O_2_-related reactive oxidative species (ROS) in *C. elegans* CL4176 following exposure to compounds and temperature shift for 26 hr. Our data showed that exposure to the *Lycoris* compounds had no effect on ROS levels in treated worms compared to non-treated worms. This result indicates that antioxidant activity was not one of the factors contributing to *Lycoris* compounds’ anti-paralysis activity in CL4176 worms.

### Cytotoxicity of *L. radiata C*ompounds in Mammalian Cells

Ultimately, we would like to screen for natural and synthetic AChE inhibitors that have potent anti-AD effects but do not induce undesirable side effects for patients. The crinine and lycorine alkaloids have been reported to have noteworthy cytotoxicity [9], [34], [35]. We tested the cytotoxicity of our *Lycoris* compounds at the highest concentration of 50 µM that was used in the *C. elegans* in FHs 74 Int cells (human fetal small intestine normal cells, ATCC # CCL-241). With the MTS proliferation test, we demonstrated that the natural haemanthidine, a crinine type of alkaloid, was the most toxic and exhibited an average (from two independent experiments) of 28% ±2.62 inhibition rate of cell proliferation compared to non-treated cells which coincided with the previous report. Our results showed that the natural galanthamine and the synthetic 1,2-Di-*O*-acetyllycorine were not toxic at all to FHs 74 Int cells at 50 µM concentration. The synthetic 1-*O*-acetyllycorine was also shown to inhibit the proliferation of FHs 74 Int cells by 12.8% ±3.98.

## Discussion

As the world population is growing older, the number of patients with Alzheimer disease (AD) is steadily increasing worldwide. With the limitations of improving the AD symptoms and the unfavorable side effects of current AD medications, it is imperative that we actively search and screen for new AD-controlling agents. We describe here the utilization of Aβ-transgenic *C. elegans* to study the inhibitory effects of two natural and two synthetic compounds from *Lycoris radiata* on paralysis of the worms caused by the Aβ toxicity. Our results showed that the natural galanthamine and haemanthidine and the synthetic 1,2-Di-*O*-acetyllycorine and 1-*O*-acetyllycorine could effectively attenuate the toxicity of Aβ expressed in transgenic CL4176 worms after the temperature shift from 16°C to 23°C (Fig. 1). Although it has been reported that the popular AD medication memantine could delay the paralysis of CL4176 nematodes [25], its effective concentration was not reported. We showed in this study that the Aβ toxicity attenuation by the *Lycoris* compounds was achieved at 10–50 µM concentrations. In comparison, 10 mM memantine had to be used to achieve similar anti-paralysis effect in CL4176 worms. Memantine, a known attenuator of N-methyl-D-aspartate (NMDA) receptors, is used to keep the degeneration of cholinergic cells in check [8]. One of our *Lycoris* compounds, galanthamine, is a known acetylcholine esterase (AChE) inhibitor that has been licensed in Europe for AD treatment. Both galanthamine and haemanthidine isolated naturally from *Lycoris* are alkaloids. Both the NMDA attenuator and AChE inhibitors are presumed to function by maintaining the healthy cholinergic status of cells. Therefore, the nearly 1000-fold lower concentration needed for *Lycoris* compounds to produce similar anti-paralysis effects in CL4176 indicates that they are much more effective than memantine in this system.

In order to evaluate the effects of *Lycoris* compounds on AChE genes, real-time quantitative RT-PCR analysis was conducted with total RNA isolated from worms that had been treated and incubated at higher temperature (23°C) for 26 hr. This time point was chosen since this is when the first symptoms of paralysis were observed and it allowed us to examine early changes in gene expression associated with the results of the treatment itself, rather than the downstream consequence of paralysis [28]. Our data demonstrated that the natural compound galanthamine was the most inhibitory to the gene expression of *ace-1* followed by the synthetic 1,2-Di-*O*-acetyllycorine and the natural compound haemanthidine (Fig. 2A). We have also shown that all of our *Lycoris* compounds could inhibit the gene expression of *ace-2* by around 3-fold. Interestingly, we did not find memantine inhibitory to the expression of *ace-1* and *ace-2*. To our knowledge, there has been no previous report of using Aβ-transgenic *C. elegans* CL4176 nematodes to study the anti-AChE activity of any AChE inhibitors. It is understood that CL4176 expresses Aβ in muscle cells of worms upon temperature up-shift and the paralysis phenotype is a result of the Aβ toxicity. However, as the creators of CL4176 pointed out that 67 genes were up-regulated and 240 genes were down-regulated in CL4176 upon temperature up-shift [28]. Since there are many mechanisms proposed for Aβ toxicity, so far there is no strong indication of which gene changes are responsible for the Aβ toxicity in CL4176 worms [28]. It is likely that the AChE inhibiting activities of our *Lycoris* compounds provide some protection to CL4176 worms against Aβ toxicity, just as memantine, an attenuator of N-methyl-D-aspartate (NMDA) receptors for cholinergic health, was found to be able to delay the paralysis of CL4176 [25]. At least, our data suggested that CL4176 *C. elegans* can be used effectively to screen compounds that are inhibitory to AChE gene expression.

Since inflammation and oxidative-induced stress are associated with Aβ-toxicity and some AChE inhibitors including galanthamine have been shown to modulate inflammation process [13], [14], we evaluated our *Lycoris* compounds for their inhibition of two *C. elegans* human homologs of TNFA1P1 genes, *F22E5.6* and *ZC239.12* and two homologs of human αB-crystallin (CRYAB), HSP16-2 (*Y46H3A.D)* and HSP16-4 (*Y46H3A.E*). These four genes have been shown to be up-regulated in CL4176 worms following the temperature up-shift [28]. Our results indicated that galanthamine and haemanthidine significantly reduced the gene expression of *F22E5.6* and 1,2-Di-*O-*acetylcorine highly significantly reduced the expression of *ZC239.12*. Haemanthidine was also found to be noticeably inhibitory to expression of the two HSP16 genes (Table 2). These data suggest that *Lycoris* compounds can both suppress AChE gene expression and are able to modulate expression of inflammation-related and stress-related genes in transgenic *C. elegans* CL4176. Since *C. elegans* really do not have the inflammation system *per se*, the benefits of *Lycoris* compounds’ anti-inflammation activity will need to be further evaluated in animal models.

Our data suggested that only galanthamine could reduce the transgene Aβ expression by 2.7-fold in CL4176 worms and the inhibitory effect on Aβ transcript level from haemanthidine, 1-*O*-acetyllycorine and 1,2-Di-*O*-acetyllycorine was negligible. However, all four *Lycoris* compounds could slightly reduce the Aβ peptide level in CL4176 worms by 6.4% to 8.5%. Since all four *Lycoris* compounds were shown to inhibit paralysis of CL4176 after temperature up-shift (Fig. 1), the slight reduction in Aβ transgene expression at both the transcript and peptide levels by these compounds may play a small role in extending the lifespan of CL4176 worms. Whether this slight Aβ reduction results directly from the AChE inhibition/cholinergic health-promoting activity of our *Lycoris* compounds needs to be further elucidated.

Additionally, we did not find anti-oxidant activity contribute to the anti-paralysis effect of the four *Lycoris* compounds. Therefore, our findings in this study strongly indicate that the anti-paralysis effects of our *Lycoris* compounds mainly result from their AChE gene inhibition and inflammation/stress-related gene modulation. However, it will be interesting to find out the inter-relationship amongst the Aβ toxicity and the cholinergic health and the lifespan extension promoted by the *Lycoris* compounds in CL4176 worms in future studies.

The present study demonstrates that human Aβ-expressing transgenic *C. elegans* CL4176 can be used effectively to screen for compounds that are inhibitory to AChE gene expression. Although we have shown that human fetal intestinal epithelial cells provide an easy system to test the cytotoxicity of AChE inhibitors, further efficacy studies need to be conducted in mouse and monkey models of Alzheimer’s disease before any new compound can be studied in human clinical trials.

## Materials and Methods

### Isolation and Synthesis of *L. radiata* Compounds

The isolation of galanthamine and haemanthidine from *L. radiata* has been reported in our previously published paper [23]. 1-*O*-Acetyllycorine [36] and 1,2-di-*O*-acetyllycorine [37] were prepared using established procedures**.**

### Paralysis Assay of Aβ-Transgenic *C. elegans* CL4176 Nematodes

Aβ-transgenic *C. elegans* CL4176 nematodes [genotype: *smg-1(cc546ts);* dvIs27] were obtained from CGC (*Caenorhabditis* Genetics Center) and maintained on NGM (Nematode Growth Medium) plates (60 mm petri dishes) at 16°C. Paralysis assays were performed according to the method of Dr. C. Link [25]. In brief, one week before initiating the paralysis assay, gravid adult worms were picked onto NGM plate spread with OP50 bacteria to lay eggs for 2–4 hr at 16°C. The gravid adults were picked off the plates and the progeny were allowed to grow for 7 days into “second day” gravid adults. One day before the initiation of the paralysis assay, NGM plates were prepared with 50 µl of fresh OP50 bacterium mixed with *Lycoris* compounds at different concentrations or 10 mM memantine and incubated at 37°C overnight. At day 1 of the initiation of the paralysis assay, the second day gravid adults were allowed to lay eggs on NGM plates containing OP50 and compounds. Approximately 50 progeny were maintained on each plate for each treatment and incubated at 16°C for two days. The worms were then incubated at 23°C and the survival rates were recorded. Worms without movement after prodding were recorded as dead.

### Quantitative Staining of Aβ with Thioflavin-T

Twenty-six or 32 hours post temperature shift from 16°C to 23°C, all the CL1476 worms for each treatment plate were collected by washing the plate with 1 ml 1× PBS (diluted from 10× PBS, Fisher) and transferred into a microfuge tube. The worms were pelleted by centrifugation at14k rpm for 2 min and sonicated with Branson Sonifier 150 at setting 2 for 15 sec ×4 times. The sonicated worms were centrifuged at 14 krpm for 2 min and the supernatant was transferred into a new tube. The concentration of total soluble protein in each sample was quantified by the Bradford method (BioRad). Equal amount of total protein from every sample was used in each independent experiment, which was divided into 3 replicates. Each replicate was mixed with 10 µl 10× PBS (Fisher) and 2 µl 1 mM thioflavin-T (Sigma) (final concentration of 20 µM) in a final volume of 100 µl. Fluorescence resulting from Aβ stained by thioflavin-T was measured by the Synergy HT Plate Reader using excitation at 440 nm and emission at 482 nm and averaged from at least three independent experiments.

### Measurement of H_2_O_2_ Levels in CL4176 *C. elegans*

Twenty-six hours after the temperature up-shift, exactly 50 CL4176 worms from each treatment plate were collected by a platinum worm-picker into 100 µl 1× PBS (diluted from 10× PBS, Fisher) containing 1% Tween-20 in a microfuge tube and sonicated with Branson Sonifier 150 at setting 2 for 15 sec ×4 times. The intracellular levels of H_2_O_2_-related reactive oxidative species (ROS) in CL4176 worms were measured as described using 2,7-dichlorofluorescein diacetate (DCF-DA, Sigma Aldrich) [21]. Briefly, sonicated worms were pipetted into the wells of a 96-well plate containing 100 µl of 1× PBS plus 100 µM DCF-DA. The final concentration of DCF-DA in the assay is 50 µM. The fluorescence was recorded by Synergy HT Plate Reader at 485 nm excitation and 545 nm emission and averaged from three independent experiments.

### Quantitative Real-Time RT-PCR (qRT-PCR) Analysis

Twenty-six hours post temperature shift from 16°C to 23°C, treated CL4176 worms were collected by washing the plate with 1 ml 1× PBS and transferred to microfuge tubes. The worms were pelleted by centrifugation at 14 krpm for 2 min. The total nematode RNA was isolated by the freeze-cracking method as described in the WormBook (http: //www.wormbook.org). Briefly, 500 µl Trizol reagent (Life Technologies) was added to each sample. The freeze-thaw was repeated three times by freezing in dry ice/ethanol bath and 5 min 37°C -incubation. After 100 µl chloroform was added, the worm suspension was then vortexed vigorously and centrifuged at 14 krpm for 3 min. Total nematode RNA in the supernatant was precipitated by ethanol, sodium acetate and centrifugation. Precipitated RNA was further purified by in gel-DNase digestion following the suggestion of Qiagen using the RNeasy column. RNA concentration and integrity was measured by a Nanodrop spectrophotometer.

cDNA was produced from total nematode RNA by reverse transcription using the High Capacity cDNA Synthesis Kit and random primers (Applied Biosystems/Life Technologies). Quantitative, real-time PCR (qPCR) was performed by Applied Biosystems SDS 7300 instrument using gene-specific forward and reverse primers (Table 1) with the following parameters: 1 cycle of 50°C for 2 min, 1 cycle of 95°C 10 min and 40 cycles of 95°C for 15 sec followed by 60°C for 1 min. The levels of relative gene expression were assayed by the 2^−ΔΔCt^ method using the *F23B2.13* gene encoding an RNA polymerase small subunit as a non-variable endogenous control.

### Cytotoxicity Assay of *Lycoris C*ompounds

Human fetal intestinal epithelial (FHs 74 Int) cells were purchased from ATCC and grown in complete Hybri-care medium (ATCC) supplemented with 30 ng/ml epidermal growth factor, 10% fetal bovine serum, 20 units/ml penicillin and 20 µg/ml streptomycin at 37°C with 5% CO_2._ 1×10^4^ FHs 74 Int cells in 100 µl medium were seeded in each well of the 96-well tissue culture plate. After overnight growth, cells were washed with 1× PBS and incubated with 100 µl complete medium containing the *Lycoris* compounds at the final concentration of 50 µM. After 24 hr incubation, wells were washed with 1× PBS, 100 µl complete medium was added to each well. MTS (Promega) reagent (20 µl) was added into each well and the plate was incubated at 37°C with 5% CO_2_ for 2 hr. The cell viability was measured at 490 nm using the Synergy HT Plate Reader. The cytotoxicity of *Lycoris* compounds was assessed as the percentage reduction of cell viability compared to the cells-only controls. All samples were tested in triplicate and the assays were repeated twice.
